# Omics Technologies in Molecular Biology

**DOI:** 10.3390/ijms27083349

**Published:** 2026-04-08

**Authors:** Andrés J. Cortés

**Affiliations:** Facultad de Ciencias Agrarias—Departamento de Ciencias Forestales, Universidad Nacional de Colombia—Sede Medellín, Medellín 050034, Colombia; ancortesv@unal.edu.co

Over the past two decades, molecular biology has witnessed unprecedented innovation driven by the emergence of omics technologies. What began as a largely mechanistic and deterministic discipline—focused on individual genes, proteins, and pathways—has evolved into an integrative, multi-system framework capable of interrogating entire biological networks simultaneously across multiple layers. Genomics, transcriptomics, proteomics, lipidomics, metabolomics, phenomics, and more recently enviromics collectively enable both bottom-up and trans-organismal understanding of biological interactions, extending across complex multicellular systems. Despite these substantial advances, persistent gaps remain, particularly when it comes to data integration and “big data” interpretation, reproducibility and scalability across different biological and ecological contexts, and the effective incorporation of emerging machine learning applications. To help bridge these gaps, this Special Issue on “Omics Technologies in Molecular Biology” seeks to explore how vast, heterogeneous datasets can be integrated into coherent, predictive frameworks that advance both conceptual understanding and the practical application of omics tools. The contributions included in this Issue reflect the diversity and promise of the field, spanning areas such as insect vector control, crop breeding, plant ecological genetics, human proteomics, and cardiovascular lipidomics. This diverse compilation of works exemplifies both the power of omics technologies and the persistent gaps that must be addressed to fully release their potential. Rather than merely summarizing the contributed studies, the editorial highlights recent developments, identifies critical remaining knowledge gaps, and outlines research perspectives that will shape a new phase of multi-omics molecular biology.

## 1. An Expanded Omics Landscape: From Single Layers to Multi-Dimensional Systems

One of the most notable modern trends reflected in this Special Issue is the transition from single-omics studies to multi-omics integration. Early omics research often focused on individual layers—such as genome-wide association studies [1] or transcriptome profiling [2]. While these approaches have offered key regulatory understanding, they frequently fell short in explaining complex polygenic phenotypes due to the multi-layered [3] and sometimes epistatic [4] nature of biological regulation [5]. Recent technological advances now enable the simultaneous screening of multiple molecular layers in an integrated manner [6]. For example, one of the contributing studies on insecticide resistance in mosquito vectors demonstrates how genomics (e.g., single nucleotide polymorphisms—SNPs, and copy number variations—CNVs), transcriptomics (gene expression changes), proteomics (enzyme activity), and metabolomics (detoxification pathways) collectively reveal the mechanistic basis of adaptive traits [7].

Specifically, the review by Bharadwaj et al. [7] examines how integrated omics approaches—genomics, transcriptomics, proteomics, and metabolomics—are advancing the understanding of insecticide resistance in mosquito vectors, a growing threat to global control of diseases such as malaria, dengue, and Zika. It highlights key resistance mechanisms, including metabolic detoxification, target-site mutations, cuticular modifications, and behavioral adaptations, and shows how omics tools have identified critical genes, regulatory pathways, and metabolic shifts underlying these processes. The review also emphasizes the value of multi-omics integration and systems biology approaches to uncover complex gene–protein–metabolite networks, enabling the discovery of biomarkers and predictive models of resistance evolution. Emerging fields such as epigenomics and microbiomics are also discussed as promising but underexplored contributors to resistance. Despite challenges such as data integration, cost, and the need for functional validation, the review concludes that multi-omics-driven studies are essential for improving surveillance and developing more effective and sustainable vector control strategies.

Similarly, another contributing study from the field of crop breeding reinforces how merging pangenomic—usually summarized through pangenome graphs [8,9,10]—and phenotypic analyses of native germplasm reveal the manner structural genomic variation, gene expression, and phenotypic traits converge to define agriculturally important characteristics such as fruit quality [11]. Specifically, this work by Vega-Muñoz and collaborators characterizes the genetic basis of fruit quality traits in 283 accessions of *Capsicum* germplasm representing all five domesticated species. Using genotyping-by-sequencing and genome-wide association studies (GWAS) based on both a reference genome and a multi-species pangenome, the authors identified up to 150 significant SNPs linked to morphological, physicochemical, and biochemical traits, revealing predominantly complex polygenic architectures. Extensive phenotypic variation in fruit morphology, texture, color, and metabolite content—including capsaicinoids and carotenoids—is observed, alongside evidence of genetic clustering consistent with species differentiation but influenced by introgression. Candidate genes associated with key metabolic pathways—particularly capsaicinoid biosynthesis, terpenoid and sterol metabolism, and cell wall modification—are also identified, providing functional insight into key trait variation. The combined use of pangenomic resources enhances the detection of novel allelic diversity compared with a single reference genome, emphasizing the value of multi-omics approaches for exploiting underutilized germplasm and advancing multi-location pre-breeding strategies aimed at improving quality and agro-industrial traits, e.g., [12,13].

Also from the plant kingdom, but this time in a plant ecological genetics context, the study by Lu et al. [14] utilizes a combination of nuclear microsatellite (SSR) markers, chloroplast DNA (cpDNA), and nuclear ribosomal DNA (nrDNA) sequences to characterize the genetic diversity and population structure in 168 samples across 16 populations of *Saussurea involucrata*, a rare medicinal herb endemic to the Tianshan Mountains in Central Asia. The team found that the understudied Eastern Tianshan populations exhibit higher genetic diversity and significant genetic differentiation from the well-documented Bayinbuluke populations. The identification of unique private haplotypes and a lack of recent genetic bottlenecks indicate historical demographic stability and a distinct evolutionary trajectory for these eastern groups, likely driven by long-term geographical isolation and local adaptation. Consequently, within a conservation genetics framework, the authors suggest that the Eastern Tianshan populations must be treated as an independent management unit (MU) to preserve their unique genetic background, thereby supporting sustainable conservation strategies for this endangered species. Therefore, the work demonstrates how omics tools can support the identification of genetic conservation gaps [15]. The proposed integrative pipeline enables the identification of novel MU that otherwise would remain inaccessible when taxonomic datasets are analyzed in isolation.

In more downstream regulatory layers, biomedical lipid droplet proteome [16] studies and untargeted serum proteomics [17] demonstrate how lipidomic and proteomic signatures can identify disease states and suggest novel diagnostic biomarkers in human genetics. As with the previous examples, these contributions highlight target applications of omics tools, extending beyond fundamental questions in molecular biology.

Specifically, the review article by Mallia and team [16] offers a comprehensive overview of how omics technologies inform the roles of lipid droplets (LDs) and their associated surface proteins in cellular lipid homeostasis and the development of cardiovascular diseases (CVDs). The authors start by detailing the dynamic life cycle of LDs—from their biogenesis at the endoplasmic reticulum and inner nuclear membrane to their degradation via lipolysis and lipophagy—highlighting how targeted omics approaches are essential for identifying the specific proteomic profiles underlying each of these processes. After examining the complex interactions between LDs and other organelles like mitochondria, the review emphasizes how dysfunctional lipid metabolism leads to pathologies such as atherosclerosis, heart failure, and myocardial lipid overload. Ultimately, the review advocates for a deeper understanding of the LD proteome through multi-omics integration to enable the discovery of novel biomarkers and therapeutic targets for the prevention, diagnosis, and treatment of cardiovascular and metabolic disorders.

Last but not least in this Special Issue (Table 1), the study by Blaha et al. [17] utilizes untargeted mass spectrometry-based serum proteomics to trace the molecular signatures across 48 individuals with Fontan circulation—a surgical palliative intervention for patients with a single functional ventricle. 

The authors Blaha et al. compare the target patients to age- and sex-matched healthy controls. The analysis quantifies 2228 proteins, identifying 124 that are differentially abundant. These candidates are organized into three functional clusters: increased extracellular matrix (ECM) organization, and decreased actin cytoskeleton organization and platelet-related pathways. Stratified analyses reveal that patients with higher clinical risk scores exhibit reduced ECM protein abundance, and uniquely elevated levels of cytochrome b5 reductase 3 (CYB5R3), a protein linked to altered redox homeostasis and cellular aging. Overall, these findings extend previous targeted studies by identifying novel potential biomarkers, and highlighting the complex systemic remodeling and metabolic adaptations inherent to Fontan physiology, which may aid in future medical risk assessment and therapeutic management.

## 2. Persistent Challenges in Multi-Omics Integration

Despite the substantial progress in data integration described above and summarized as part of this Special Issue, merged data interpretation across broader omics layers remains one of the most significant bottlenecks in modern molecular biology research. The complexity arises not only from the volume of data but also from its heterogeneity [18]. Different omics platforms generate datasets at distinct scales, levels of accuracy, and feature types, complicating their interpretation [19].

While the contributions in this Special Issue demonstrate that high-resolution datasets are now readily obtainable and partly integrated, the studies also acknowledge some of the above challenges. For instance, when it comes to cross-platform compatibility, integrating genomic, transcriptomic, proteomic, and metabolomic data requires harmonization of data formats and normalization strategies for quantitative data [20]. Discrepancies in data resolution and coverage can lead to incomplete or biased interpretations [21]. This gap limits comprehensive multi-layer functional interpretation. Even when integration is technically feasible, translating multi-omics data into biological meaning remains a paramount challenge [22]. For instance, differential gene expression does not always correlate with protein abundance or metabolic activity, emphasizing the need for cross-validation [23]. In turn, the inherent network complexity in biological systems is governed by highly interconnected metabolic pathways [24]. Identifying causal relationships within these networks, rather than mere correlations, requires more controlled experimental approaches that are difficult to scale to larger non-model populations [25].

Addressing these limitations requires more standardized pipelines across omics sub-disciplines [18], improved open-access, findable, and traceable data-sharing frameworks across specialties and institutions [26,27,28], as well as the development of integrative analytical and predictive models capable of capturing non-linear and context-dependent interactions [29]. Reaching this level of consolidation requires acknowledging the big data dilemma—more data is not sufficient without the analytical frameworks to handle it. Specifically, omics technologies generate massive datasets that demand scalable computational infrastructure and robust analytical methods [30]. As omics profiling costs decrease and throughput increases, the field is transitioning from a data acquisition bottleneck to data overabundance. However, increased data volume has not automatically translated into a more precise understanding of the underlying biological systems. This is because high-throughput data often contain substantial technical and biological noise, a trade-off referred to as the signal-to-noise ratio. Moreover, reproducibility still intrinsically varies across omics platforms, laboratories, and analytical pipelines, thereby compromising overall replicability. Even when accuracy and reproducibility issues are accounted for, access to the computational resources and expertise required to handle large-scale datasets are not evenly guaranteed.

Fortunately, in the last decade, artificial intelligence (AI) and machine learning (ML) have emerged as powerful tools for integrating and analyzing complex omics datasets, bridging some of the gaps just described [31]. Furthermore, their ability to identify patterns in high-dimensional data makes them particularly well-suited for multi-omics integration. In the context of this Special Issue, ML approaches are now assisting with the prediction of phenotypic traits from phenomic [32,33], enviromics [34], and genomic data in crop breeding, forestry, and livestock [35,36,37,38,39], population stratification and clustering in ecological genetics [40,41,42,43], and early disease detection [44,45]—as well as subtype and stage classification—based on omics biomarkers. Despite these promises of modern AI and ML algorithms, they still face additional limitations, too. For instance, ML models require large, well-annotated datasets for training, yet in many cases such datasets are still limited or imbalanced across omics layers [30,31]. As a result, some models are trained on overly specific datasets, limiting their ability to generalize across contrasting populations, environments, and experimental conditions. Moreover, many ML models, particularly deep learning approaches [46,47,48,49,50], function as “black boxes”, making it difficult to interpret the biological relevance of their predictions. To fully harness ML-assisted omics, robust cross-dataset validation, explainable AI approaches, and integration with mechanistic models are required.

## 3. Perspectives

Given the persistent gaps in omics data integration outlined in the previous section, some short-term avenues to bridge them can be identified. First, future studies must move beyond parallel, individualized analyses toward fully integrated and reproducible frameworks that capture interactions across omics molecular layers. This will require cross-disciplinary collaboration [51], more standardized, scalable, and sharable data formats [26,27,28], and innovative computational frameworks [29]. Among the latter, developing methods capable of distinguishing causation from correlation—a long-standing debate in molecular evolution [52]—would be essential across multi-omics networks to fully comprehend the underlying biological processes. Such methods must rely on open data repositories [28], standardized pipelines [29], and reproducible workflows [53] to ensure reliability and comparability across populations and study systems. Additionally, as discussed above, explainable ML and more interpretable AI models can further enhance the biological relevance and downstream application of computational predictions.

While the analytical advancements are urgent and likely to continue progressing at an exponential pace in the next decade, technical considerations should not be overlooked. For instance, emerging technologies capable of capturing molecular data at single-cell resolution [54], and across various spatial, tissue, and temporal resolutions, will provide an unprecedented understanding of regulatory and activation cascades across cells, organs, and timelines. Technologies will also continue improving in their high-throughput capacity and data quality, likely blurring the antagonistic dichotomy between data volume and accuracy. In the genomic field, the trade-off is evident when comparing the last generation of HiFi PacBio sequencing technology and the Oxford Nanopore [55,56], the latter being able to capture longer sequences, up to Mb, while compromising base call precision. The phenomic field is perhaps the one that already demonstrates a case in which the contradiction has been effectively buffered. Conventional phenotyping is highly prone to the human observer error, introducing non-systematic biases across trait datasets. Instead, phenomics have not only improved the speed at which phenotypes can be recorded, but also their precision [57,58,59]. It remains to be seen whether other omics sub-disciplines will be able to accomplish similar breakthroughs in the volume vs. precision dilemma in the years to come.

Another major opportunity arises with the recognition of a new omics dimension, referred to in recent years as enviromics [60]. It is one of the most underexplored yet critical facets of modern omics research, which aims to assess the influence of environmental gradients on molecular systems [61]. Particularly, incorporating environmental data into multi-omics analyses will provide a more compelling view of biological systems and their plastic and adaptive responses across exogenous contexts [62]. Moreover, biological processes do not occur in isolation; instead, they are continuously shaped by environmental factors such as ecological interactions [63], temperature, pollutants, diet, among many others. The importance of enviromics is already tangibly evident in some of the contributions to this Special Issue. For instance, in mosquito vectors, environmental exposure to insecticides drives the evolution of resistance mechanisms at genomic and metabolic levels. Likewise, in plant systems, environmental gradients influence crop responses, as well as genomic diversity and phenotypic adaptation [64,65]. In human health, environmental factors and diet modulate proteomic and metabolic profiles, influencing disease progression. Despite its transversal importance, enviromics remains poorly integrated into multi-omics frameworks, opening significant opportunities for future research [66]. Among these, multi-population as well as multi-locality studies would help capture environmental variability. In turn, to integrate environmental metadata with molecular datasets, novel models linking environmental gradients to molecular and phenotypic features are needed [67]. Once enviromics is acknowledged and integrated within systems biology frameworks [68], we will able to achieve a more holistic understanding of how intrinsic omics determinism interacts with the nurture of exogeneous environmental contexts in shaping phenotypes and diseases. Only then can enviromics truly bridge molecular omics with the environment contexts in which biological systems are intrinsically embedded.

**Figure 1:**
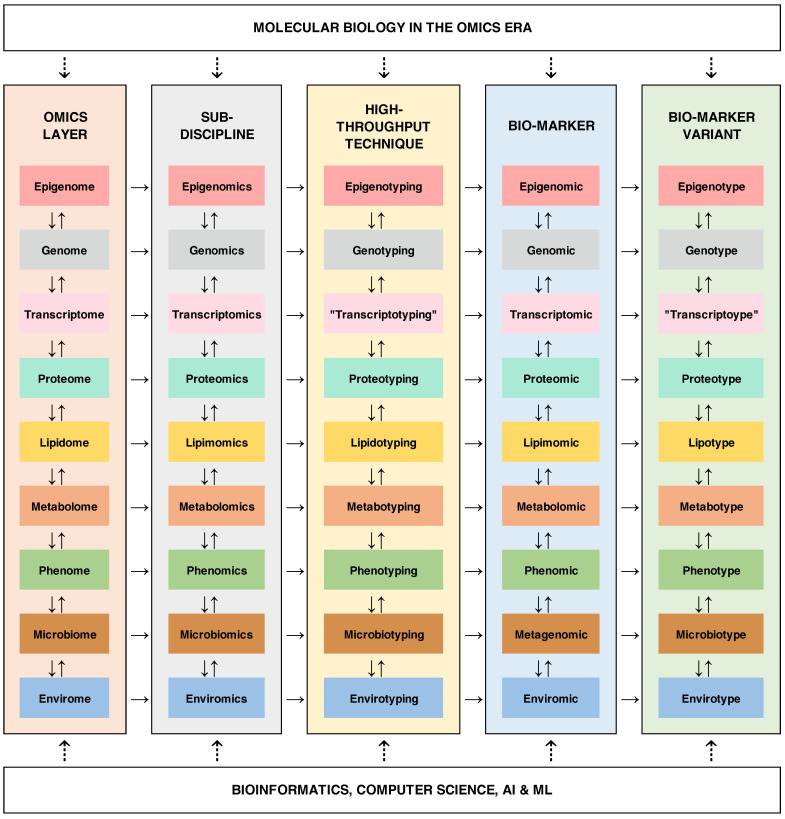
Conceptual framework linking multi-omics layers, analytical sub-disciplines, and derived biomarker variants in the omics era. This figure illustrates the hierarchical and integrative structure of biological organization across multiple omics layers, ranging from molecular to environmental scales. Each row represents a distinct layer of biological information—epigenome [85], genome [86,87], transcriptome [88], metabolome [89,90,91], lipidome [92,93], proteome [94], phenome [95,96], microbiome [97] (*s.l.* metagenomics [98]), and envirome—progressing from regulatory and genetic upstream determinants to organismal traits and environmental interactions. Across columns, the figure reinforces the conceptual and methodological continuum connecting (*i*) the biological layer (e.g., genome), (*ii*) its corresponding analytical discipline (e.g., genomics), (*iii*) modern high-throughput characterization approaches (e.g., genotyping), (*iv*) the type of biomarkers generated (e.g., genomic), and (*v*) the resulting marker variants (e.g., genotype). This structure emphasizes how each omics field produces distinct but comparable data types that can be standardized as “-types” (e.g., genotype, phenotype, metabolotype), facilitating much-needed integrative analyses. Bidirectional arrows are not meant to be pairwise but multi-layer, indicating the feedbacks and interactions among omics layers, highlighting that biological systems are not strictly linear but instead involve complex regulatory networks across scales. For example, according to the central dogma *status quo*, genomic variation influences transcriptomic profiles, which in turn affect proteomic and metabolomic levels, ultimately shaping phenotypes. However, reverse regulatory interconnections are known to shape expression at multiple levels, too. The lower section reinforces the critical contribution of bioinformatics, computer science, artificial intelligence, and machine learning in processing, integrating, and interpreting these large-scale multi-omics datasets. Together, this framework summarizes not only the modern omics taxonomy (i.e., quotation marks highlight unconventional terminology so far), but also the transition toward systems-level understanding in modern biology, where multi-omics integration is essential for deciphering complex polygenic and ominigenic traits [99] and context-dependent adaptive processes.

Lastly, besides boosting multiple omics integration (Figure 1), it is essential to recognize the need for the field to move beyond understanding biological mechanisms toward harnessing their applications, embracing the translational impact of omics research [22,69,70]. Regarding the studies included in this Special Issue, it is possible to identify some promising avenues for more applied uses of omics profiling. For instance, multi-omics analyses of insecticide resistance in mosquito vectors not only enable the understanding of underlying adaptive mechanisms but also support the development of targeted vector control strategies [71,72,73]. Similarly, in the context of modern agricultural and food security pressures, pangenomic, phenomic, and, eventually, pan-omic analyses of crop germplasm would provide a baseline [74,75] for more precise “omic-estimated breeding values” (OEBVs) and omic predictive selection [76,77], enabling the development of varieties with improved yield, quality, and resilience in shorter timeframes and with greater precision [78]. Extending the application of multi-omics technologies into the ecological genetics field would allow for a more accurate identification and prioritization of cryptic conservation targets [79] in endangered species as part of worldwide efforts to preserve biodiversity and its ecosystem services [80,81,82,83]. In the medical diagnosis sector, lipidomic and proteomic profiling in systemic and cardiovascular diseases reveals molecular signatures that can inform—together with “poly-omic” risk scores [84]—diagnosis, therapeutic strategies, and prognosis. In the long term, translating omics insights into concrete uses would broaden the impact of omics tools across disciplines. However, as with any technology, the translation of omics data into real-world solutions requires innovation, trans-disciplinary collaboration, robust validation, and consideration of societal and ethical implications, particularly those related to equitable access and data protection.

## 4. Conclusions

The contributions to this Special Issue have offered an impressive overview of how modern omics technologies are shaping the field of molecular biology. From elucidating adaptive mechanisms in ecological systems to uncovering genetic diversity in plants and omics signatures in human diseases, omics approaches are redefining our understanding of life. However, omics research still faces significant opportunities for improvements. The current challenges in data integration, scalability, interpretability, and environmental contextualization must be addressed to fully release the potential of multi-omics research. The field is now transitioning from data generation toward the integration of data into predictive, reliable, and applicable frameworks, providing fertile ground for modern AI and ML technologies. Computational innovation must continue to evolve in parallel with interdisciplinary collaboration, which must be fostered across the community through open data repositories and standardized analytical workflows. This Special Issue offers an initial step toward that goal, providing a platform for the dissemination and discussion of omics research advancements.

## Figures and Tables

**Table 1:** Summary of the five multi-species multi-omics study compilation as part of Int. J. Mol. Sci.’s Special Issue on “Omics Technologies in Molecular Biology”. The table summarizes in each case the most-relevant research goal, sampling, and finding from an omics perspective.

Species Group *	Study Goal	Sampling	Omics Technologies	Omics-Related Key Findings	Reference
Mosquito vectors (*Aedes* spp., *Anopheles* spp., *Culex* spp.)	Understand insecticide resistance in mosquito vectors	Review of literature	Genomics, transcriptomics, proteomics, and metabolomics	Multi-omics improve surveillance and control strategies development	Bharadwaj et al. [7]
Chilies (*Capsicum* spp.)	Characterize the genetic basis of fruit quality traits in *Capsicum*	A total of 283 genebank accessions screened for 18 fruit traits	Pangenomics and 27,906 SNPs	Pangenomic resources leverage germplasm diversity for breeding	Vega-Muñoz et al. [11]
Endemic medicinal herb (*Saussurea* *involucrata*)	Assess genetic structure and diversity in endemic populations	A total 168 samples across 16 populations in Tianshan Mountains	Microsatellites (SSR) markers, and cpDNA and nrDNA sequences	Genetic screening identifies cryptic conservation management units	Lu et al. [14]
Humans (*Homo sapiens*)	Assess the effect of lipid droplets (LDs) in cardiovascular diseases (CVDs)	Review of literature	Lipidomics and lipid droplet proteome	LD proteome offers novel biomarkers and therapeutic targets for CVDs	Mallia et al. [16]
Humans (*Homo sapiens*)	Trace the molecular signatures of Fontan circulation (FC)	A total of 48 patients with FC, and age- and sex-matched controls	Untargeted mass spectrometry-based serum proteomics	Omics aid in medical risk assessment and Fontan circulation management	Blaha et al. [17]

* The table is sorted top-down by species (i.e., animal, plant and human model), and scope (i.e., review vs. case studies).

## Data Availability

The original articles [7,11,14,16,17] compiled as part of the Special Issue “Omics Technologies in Molecular Biology” are available here https://www.mdpi.com/journal/ijms/special_issues/5LJN87VERF, as accessed on 19 March 2026.
